# Mr. Thomas, on Dr. Bellamy's Case of Diseased Bladder

**Published:** 1807-01

**Authors:** Charles Thomas

**Affiliations:** H. M. Ship, Resolue, Plymouth


### *To the Editors of the Medical and Physical Journal.*

Gentlemen,

It is not without reluctance I feel myself impelled to enter the field of controversy with two of your Correspondents, who in the Medical and Physical Journal of last month, have commented with no little severity, on a communication from Dr. Bellamy, published in the preceding number.

Each has gone into a close and rigid examination of the case; but whether with the success which might naturally be expected from a tone so confident, it is not for me, but the whole medical world to determine. I mean not to dogmatize, but only to state an opinion, when I declare, that in my estimation, their triumph is far from being complete; an opinion I hope to substantiate by proof pertinent, and convincing.

I am as anxious as these Gentlemen, that every medical opinion and practice, which is exposed to the suspicion of being founded on error, should undergo the ordeal of strict, strict, unbiassed criticism; so that nothing of doubtful value, on subjects in which the welfare of our fellow-creatures is so deeply involved, may have the stamp of authority, and thus pass current.

Any acquaintance I have with the Author of the paper, which has drawn forth their disapprobation, shall not, I faithfully promise them, prevent me from exercising a spirit of impartiality in the examination of it, and their remarks. That I possess Dr. Bellamy's acquaintance I readily avow, and reflect on the advantages derivable from it with satisfaction. Though stigmatized in one page of your Journal as a mere "naval practitioner," and described in another as perhaps "experienced and well informed, but ignorant of the improvements of modern Surgery;" yet I verily believe I risk no hasty unfounded declaration, when I pronounce his professional attainments to be such as would bear the fullest comparison with those of either of his opponents. Not very many have had more experience. It has been obtained at periods of distinguished glory to our country, as well as on various other occasions; and if he has not derived some profit from such advantages, he is certainly a great deal more stupid than I take him to be.

While I give my suffrage thus freely and favourably to Dr. Bellamy's professional character, I readily admit that his treatment of the case lately published is not consonant with his usual judgment, and open to objection. It is even granted, that the two gentlemen, who have canvased that treatment and his observations, have convicted him of error. And I agree so far with Mr. Dawson in allowing, that on the second, or "third day at farthest," the proper and only indication was, "to lessen inflammation by a strict observance of the antiphlogistic regimen." But though I concede thus much, I still must be permitted to contend, that there are several collateral circumstances, which ought to weigh so much in extenuation as to subject him to a far less degree of censure than has been heaped on him.

It must be confessed, that the bare form and phraseology of Dr. Bellamy's paper are not calculated to conciliate the good opinion of its readers. The whole seems to be a literal transcript of the book, which is usually kept by Surgeons of the Navy, and where their daily practice is hastily noted down. In a large ship, the number of patients being generally from twenty to thirty, we cannot reasonably expect to meet with much elegance or perspicuity of diction in a volume of this nature. But though such "a plain, unvarnished tale may not gratify the fastidious ear of the critic, the lover of truth will approve of this rigid adherence to her sacred dictates, and praise the author for the ingenuousness he has shewn, in keeping back no circumstance or fact connected with a faithful history of the case.

This case, it is evident, was one of stricture. While no inflammatory symptom appeared, the practice at first adopted is perfectly justifiable; a practice to which I have resorted, and would again, under similar circumstances. The efficacy of mercury, in promoting the absorption of diseased deposit in various parts of the system, is known to every practitioner; and there can be no doubt, that it has often produced this beneficial effect in those contiguous to the urethra as well as in the liver. Therefore the treatment for the "two first days," should have been exempted from the animadversions which have been bestowed on it. The termination of the case did not prove "the swelling to be no chronic enlargement," as Mr. Dawson confidently asserts. It might with propriety have been so termed at the commencement of Doctor Bellamy's statement, when we consider how long it had existed in a more or less evident form, and that then no sign of inflammation was present; for those who think with me, will not be much disposed to assent to the reality of such an occurrence, by any "demonstrable proof" to be discovered at that period of the disease.

The gentlemen, who reprobate the whole treatment, seem to have forgotten one material point. The same appearances occurred six months before, and were then successfully managed "by the very same means." Such a result, and so recent, was surely enough to wed the author to it, so far at least as to give it another trial; and even to carry it rather beyond what a rational theory would authorise, provided he had not the all-powerful support of fact and experience on his side.

Mr. Chalmers expected a previous history of the case from the time the patient "first complained." Such information, I should conceive, would not have been at all interesting; as we can easily imagine, what must have been the valetudinary state of a man, whose urethra at one part was always nearly obliterated. From the time he was regularly under the surgeon's care, six months before he had probably been often "complaining;" and might have felt as much uneasiness as on the day when attendance tendance on him recommenced. This gentleman declares Dr. Bellamy to have betrayed a want of anatomical knowledge; but from what part of his publication can such an inference be legitimately drawn?

Mr. Dawson thinks it not sufficient to condemn the whole medical treatment, but he labours also to fix on Dr. Bellamy a charge, which, if proved, would reflect greatly on his prudence and skill as an operator. To establish this point, several expressions, unguardedly employed, but of ambiguous and relative import, are brought forward. The bare production of such terms as "considerable trouble" and "some force," is, it seems, to be received by us as "confirmations strong." But let us look for evidence of such an unfortunate event as a rupture of the urinary canal by the mismanagement of the surgeon, not in mere words of such a nature, but by considering the circumstances as they regularly occurred, which are supposed to have produced it.

Whenever this accident happens, I presume, it is the "immediate," and not the "remote" consequence of violence offered to the urethra; that it shews itself the moment the catheter is thrust through the canal, or the first time after, when the patient attempts to make water; but is never brought on "gradually" from a sloughing, induced by such violence. If the position I have just laid down be correct, all this gentleman's surmises on this part of the subject fall to the ground.

Let us inquire then what were the real circumstances, connected with such a supposed accident, on the evening of the 29th of February; the day on which it was first discovered by the surgeon, that the urine was discharged per perinæum? The catheter was last introduced on the evening of the 27th, or "forty-eight" hours before such a discovery. He examined at the usual time on the 28th, "without the catheter;" and we can reasonably infer nothing else but that he did the same on the 29th.—It is plain, Dr. Bellamy's method is to narrate the occurrences of the preceding twenty-four hours every evening. When therefore we read on the 30th "by close questioning, he says, he has felt "*to-day*", when making water, a sense of trickling and heat, this must be understood of no other day but that "then present." Such "questioning" was perfectly natural, in order that every possible confirmation might be had, about the existence of this preternatural opening. The catheter, it is perfectly clear, was "then" passed, that is, on the 30th; and after the urethra had been

 perhaps perhaps twelve hours in this disorganized state, but not before, the circumstance takes place, which has been so emphatically noticed in these words, "The obstructions may be felt breaking down under it." What gave this idea of obstructions, was in all probability, nothing more than coagula, with which in this condition of extensive disease, we may suppose the urethra to have been impacted. On this point then at least, I think we are justified in assuming, that the perspicacity of this Gentleman, which would lead him to look beyond the ken of vulgar minds, has hurried him away from the boundaries of sober consideration, into the regions of rash and premature conjecture; as the Readers of your Journal must recollect it did in another instance not very long since.*

An observation from Mr. Chalmers, not at all relevant to the question under discussion, and certainly not worthy of that liberality of sentiment, which I am bound to suppose him the possessor of, must not pass unnoticed; and here, as well as in the other parts of this letter, I hope, the "characteristic lameness" of my brethren will not be observable. There is an ambiguity in this Gentleman's language, which leaves the reader in doubt, whether he ever actually in his professional capacity belonged to the Navy, or not.—If he did, and had served in it half a century, he could not have acquired information enough to have formed such a sweeping and comprehensive judgment, as he has given of the Naval Medical Profession. They are stigmatized almost "in toto." The exceptions are only "some few." But restricted in this respect, as it is fair to conclude he must be, how can he be supposed to know scarcely any thing of a body of men, six hundred in number, and so situated, that in the course of his life he may not have met with a dozen of them?—Is mere hear-say sufficient ground for such an opinion? or is it fair, that the casual discovery of a single incompetent, or otherwise disgraceful character, should brand with disrespect and contempt all, let them be ever so many, who chance to be employed in the same avocations?—In all branches of the Medical Profession, as well as every other, an unworthy member is occasionally found. This may happen among "private Practitioners" and in the "Army" as well as in the "Navy;" but let it be found whenever it may, forms

---

* Medical and Physical Journal for July, 1805, p. 17.—Id. for August, 1805, p. 155.—Id. for Sept. 1805, p. 242.—Id. for Novem. 1806, p. 416.

forms a miserable foundation for such wholesale condemnation. As to the medical men of the Navy, while they are conscious of not deserving less than other members of the profession, and possessing, as they do, the public and unequivocal approbation of a Currie, a Trotter, and a Blane, they can remain perfectly regardless of such impotent expedients, to disparage and degrade them.

The Gentlemen, whose papers I have attempted to answer, however defective they may find me in controversial qualifications, will, I trust, have no reason to complain, that I have been at all wanting in that personal respect they strongly claim from me. In pointing out the difference between us, I have studiously avoided the very semblance of offence; and courted that temper, and expression of it, which so well becomes all, who are engaged in the cause of humanity and liberal science. And should I ever discover, that a failure of this kind from me, remains to stain the pages of the Medical and Physical Journal, I sincerely assure them, it would be viewed by me with feelings of the most painful regret.

I am, &c.

CHARLES THOMAS.

*H. M. Ship, Resolue,*
*Plymouth, Sept.* 12, 1806.

---

### *To the Editors of the Medical and Physical Journal.*

Gentlemen,

